# Natural Peptides Inducing Cancer Cell Death: Mechanisms and Properties of Specific Candidates for Cancer Therapeutics

**DOI:** 10.3390/molecules26247453

**Published:** 2021-12-09

**Authors:** Plinio A. Trinidad-Calderón, Carlos Daniel Varela-Chinchilla, Silverio García-Lara

**Affiliations:** Tecnológico de Monterrey, School of Engineering, and Sciences, Avenida Eugenio Garza Sada 2501, Monterrey 64849, Nuevo León, Mexico; plinio.trinidad@tec.mx (P.A.T.-C.); carlos.varela.ch@gmail.com (C.D.V.-C.)

**Keywords:** peptide, cancer, cell, death, therapeutics, mechanism, apoptosis, membrane, model

## Abstract

Nowadays, cancer has become the second highest leading cause of death, and it is expected to continue to affect the population in forthcoming years. Additionally, treatment options will become less accessible to the public as cases continue to grow and disease mechanisms expand. Hence, specific candidates with confirmed anticancer effects are required to develop new drugs. Among the novel therapeutic options, proteins are considered a relevant source, given that they have bioactive peptides encrypted within their sequences. These bioactive peptides, which are molecules consisting of 2–50 amino acids, have specific activities when administered, producing anticancer effects. Current databases report the effects of peptides. However, uncertainty is found when their molecular mechanisms are investigated. Furthermore, analyses addressing their interaction networks or their directly implicated mechanisms are needed to elucidate their effects on cancer cells entirely. Therefore, relevant peptides considered as candidates for cancer therapeutics with specific sequences and known anticancer mechanisms were accurately reviewed. Likewise, those features which turn certain peptides into candidates and the mechanisms by which peptides mediate tumor cell death were highlighted. This information will make robust the knowledge of these candidate peptides with recognized mechanisms and enhance their non-toxic capacity in relation to healthy cells and further avoid cell resistance.

## 1. Introduction

The development of novel therapeutic agents or strategies targeting cancer has become urgent [1]. Specifically, recent oncological therapeutics have been addressed by multiple approaches [2]. Commonly, surgery, radiation, and systemic treatment, such as chemotherapy and immunotherapy, are combined, among other things [3]. Specifically, newer cancer therapeutics have focused on targeting drug delivery and enhancing selective high cytotoxicity against cancer cells, contrasting with the broad effects of conventional therapy [4].

The recently proposed alternatives are notably represented by hybrid molecules coupled with conventional chemotherapy drugs [5], computer-aided drug discovery [6], and bioactive peptides [7]. Precisely, peptides have shown a trend for performing as therapeutics against cancer cells when obtained from native proteins [8]. Based on this, there are diverse databases, such as CancerPPD [9], SATPdb [10], or THPdb [11], or sequence-based peptide predictors, such as iACP [12] or mACPpred [13], suggesting possible applications and bioactivities of peptides [14]. Nevertheless, even when their effects on cancerous cells are well known, the specific mechanisms by which most peptides act remain undetermined [15].

Therefore, in this review, relevant peptides with a known sequence and specific anticancer mechanisms whose application has reached pathway-level studies and are candidates for anticancer therapeutics were discussed. Moreover, peptides’ characteristics that make them candidates and the general mechanisms by which cancer cell death is mediated by peptides were presented.

### Methodology Used in Literature Research

Scopus and Google Scholar databases were searched for the keywords anticancer, linear, natural, and peptides. The timespan for literature research was established from 2016 to date. Original and review articles having the specified keywords were included and indistinctly revised for content. Likewise, those articles having the keyword analogs, antibody, antioxidant, conjugate, cyclic, depsipeptide, fraction, nano, proteomic, saccharide, secondary, synthetic, and vaccine were excluded from the analysis of this review.

## 2. Properties of Therapeutic Anticancer Peptides

Recently, peptide definition has come into a debate, with the Food and Drug Administration defining a peptide as any polymer composed of 40 or fewer amino acids [16] and the European Medicines Agency considering them as small molecules if created chemically or as biological entities if extracted from natural sources [17]. Thus, their classification remains contested [18]. Here, peptides were considered molecules consisting of amino acids linked by peptide bonds, ranging from 2 to 50 residues [19].

There are wide varieties of bioactive peptides in nature, and nearly thousands have been discovered [20]. Due to their innovative pharmacological profile and intrinsic properties, peptides represent a match point for novel therapeutics design [21]. The evidence from different studies addressing the structure and activity of both natural and synthetic anticancer peptides claims that various factors are responsible for their potential pharmacological applications [22,23,24]. Thus, in this section, the most relevant features of peptides that make them candidates for cancer therapeutics are presented.

### 2.1. Amino Acid Composition

Regarding peptide structures, glycine and arginine amino acid residues are prominent in peptides targeting cancer cells [25]. Furthermore, Cys, Gly, Ile, Lys, and Trp are found in various locations of anticancer peptides [26]. Particularly, glycine, because of its structural role (β-turns) and cyclization potential, and arginine for its role in cancer therapeutics are crucial elements for the structure and bioactivity of these peptides in toto [27].

Comparatively, arginine, a positively charged basic amino acid [28], has the capacity to enhance the permeability of biological membranes due to the guanidium group found in its side chain [29]. This functional group triggers the interaction between arginine and water or phosphate groups in membrane phospholipids, thus promoting the formation of hydrogen bonds that can destabilize, disrupt, or permeabilize membranes [28,29]. Remarkably, the cell-penetrating efficacy of Arg-rich peptides relies on the number of arginine residues in the peptide sequence [30].

### 2.2. Amphipathicity

Amphipathicity is defined as the ability to survive under hydrophobic and hydrophilic conditions [31]. In this regard, certain peptides have a cationic NH_2_-terminal forming an amphipathic α-helix, which can interact with anionic elements of the cell membrane and is responsible for mediating cytotoxic effects with cancer cells [32]. Moreover, these α-helical peptides share the characteristic that they possess an amphipathic conformation with the nonpolar and polar face in a hydrophobic environment [33].

### 2.3. Hydrophobicity

Anticancer peptides have known requirements for their activity, including being moderately hydrophobic [34], the molecular feature of being repelled by water [35]. Specifically, the hydrophobic content of anticancer peptides is about 30% but can be higher in some cases [36]. Moreover, hydrophobic amino acids are found in positions from 45–225°, and polar amino acids are found in the other helix face [37]. Particularly, hydrophobicity has been observed to influence the bioavailability and transport of bioactive peptides [38]. Likewise, most α-helical anticancer peptides have a range of 40–60% hydrophobic amino acids in their composition [37].

### 2.4. Net Charge

Anticancer peptides have been shown to be generally cationic when found in neutral pH, with their charge varying from +2 to +9 [36]. Respectively, many studies have indicated that a greater positive net charge increases peptide potency and variably affects cell selectivity [37]. Specifically, the overall positive net charge is given by arginine and lysine amino acid residues [36]. Additionally, the net charge affects peptide bioavailability as carrier transport have a higher affinity for neutral peptides, whereas paracellular transport preferentially transports oligopeptides with net negative charge [38]. Furthermore, peptides with charged functional groups have reduced intestinal absorption at different sites, resulting in reduced bioavailability [11].

### 2.5. Secondary Structure in Membrane

This particular feature refers to recurrent arrangements in the space of sequential amino acid residues along a peptide chain [39]. The largest recognized group of secondary structures in anticancer peptides is the α-helical (approximately 30%) [37]. Although this secondary structure is the most common, peptides may also adopt a β-sheet or a linear structure [36]. Correspondingly, the secondary structure is generally inducible by the interaction with a lipid bilayer or a water mixture, showing that peptides have adaptive conformations related to their anticancer and antimicrobial properties [37].

### 2.6. Spatial Structure

Peptides with specific spatial conformations have shown higher activity than linear chemically synthesized peptides [40]. Interestingly, those peptides with an α-helical structure may have clear hydrophilic and hydrophobic surfaces or have a concentration of amino acids in the N-terminal and C-terminal to have distinct hydrophilic and hydrophobic sides, both of which allow binding and interactions with the lipid membrane [20]. Moreover, it has been observed that peptide stability may depend on its structural conformation [40]. Specifically, studies have argued that the α-helical structure has a more stable structure and is responsible for most of the anticancer activity of some peptides [20].

### 2.7. Oligomerization Ability

Oligomerization is a fundamental feature of peptides [41] related to their structure and function and influences their binding affinity [42]. As previously mentioned, peptides usually have a hydrophobic C-terminal that has been proposed as the facilitator for peptide entry into cells; thus, enabling oligomerization and successively pore formation and cell death [32]. Furthermore, studies have shown that dimeric and tetrameric peptides display a higher cytotoxic effect on cancer cells [43], proposing that oligomerization improves anticancer activity [44].

## 3. Characteristics of Cancerous Cells Making Them Susceptible to Peptides

Peptides targeting cancer cells are of prominent interest [45], mainly because they bind in a non-specific manner to negatively charged structures, which are both exclusively and homogenously displayed by cancer cells [46,47]. Such targets are mainly represented by phospholipids, such as phosphatidylserine (PS), which are secluded in the inner side of the plasmatic membrane in normal cells, allowing to increase specificity [47,48].

Additional characteristics, such as cholesterol content and presence of microvilli on cancer cell surface, allow to enhance susceptibility to peptides and promote selective cytotoxicity as well [49]. Therefore, this section elaborates on the significant role of these cell features.

### 3.1. Negative Charge

Nowadays, different studies still claim that cancer cells possess a negatively charged cell surface [45]. Specifically, the negative charge of cell surface mainly derives from (1) overexpression of PS (9%), (2) proteoglycans side chains in the form of heparin sulfate, (3) presence of repeated regions of O-glycosylation on membrane mucines, and (4) high levels of phosphatidylethanolamine [50,51]. 

Remarkably, high levels of ROS and hypoxia, both modifying factors of tumor microenvironment, are able to induce dysregulation in membrane phospholipids [52]. Hence, cancer cells tend to lose their asymmetric phospholipid distribution between layers of plasmatic membrane and, thus, expose PS outside [53].

### 3.2. Cholesterol Content

The fluidity and stiffness of cancer cells usually get affected when peptides are internalized into the hydrophobic layer of plasmatic membranes which, consequently, favor cell lysis [54]. Thus, further research remains needed to accurately determine the role of plasmatic membrane fluidity, mainly because the high levels of membranal cholesterol in lipid rafts can reduce the lytic action of peptides toward cancer cells [45,55]. 

Until now, cancer cell membranes, e.g., those from leukemia and lung cancer cells, are frequently more fluidic than those from healthy cells because of a lower cholesterol level [56]. Counterintuitively, a reversed trend has been observed in breast and prostate cancer cells [45]. 

### 3.3. Microvilli

A high number of microvilli both enhances surface contact area and increases the attraction of peptides to cancer cells [50,57], in contrast to healthy cell membranes [58]. Further, the irregular shape and varied size of microvilli are reported to alter cell adhesion, extracellular communication with the microenvironment, and the receptor accessibility of cancer cells in response to the exposition to cationic macromolecules, e.g., peptides [45]. 

## 4. Cell Death of Cancerous Cells Mediated by Peptides

Anticancer peptides can display effects such as cell death against cancer cells [11]. Accordingly, the different time dependence of cell death induction by peptides indicates the existence of other mechanisms [59] that also seem to depend on the presence of anionic lipids [60]. In this section, the general mechanisms by which peptides perform cell death are comprehensively described as follows: (1) membrane interaction causing disruption or micellization of the cytoplasmic membrane through pore formation [41], (2) necrosis, and (3) apoptosis induction through membrane interactions [61] or entering the cell to reach a mitochondrial target [60].

### 4.1. Disruption of Cell Membrane

Pore-forming proteins are characterized by being water-soluble and able to insert themselves into lipid bilayers [62]. Specifically, different interactions of peptides with the lipid membrane of cancer cells have been documented as the secondary structure of peptides allows them to interact with the negatively charged membrane of neoplastic cells [37].

#### 4.1.1. Transient Pore Formation

Pore formation by peptides can be found naturally in bacterial toxin function, viral infection, apoptosis, and innate immunity, hence performing in medical applications [63,64]. Transient pores occur due to membrane leaking immediately after the exposition to peptides, with most leakage happening during this time, rapidly slowing afterward, and eventually attaining a plateau with incomplete leakage [65].

#### 4.1.2. Membrane Disruption

This phenomenon refers to membrane destabilization through several modes of lysis, which may include pore formation, lipid disorganization, or mechanical stress [66,67]. Frequently cited models describing this interaction are the barrel-stave pore, toroidal pore, carpet, and the detergent-like and unifying Shai–Huang–Matsuzaki models [37,68,69].

The most recognized models for pore structure are the barrel-stave pores, defined as a cylindrical pore lined by peptides, and the toroidal pore, where two membrane leaflets bend and join themselves [63]. Regarding toroidal pores, these pores can change dynamically, demonstrating a multilevel signal when measuring a transmembrane current [70].

### 4.2. Necrosis and Apoptosis

Currently, the classification of programmed cell death includes 11 cell death mechanisms, with apoptosis among them [71]. Necrosis is excluded from such classification because of its unregulated nature, although recent scientific evidence claims that necrosis can be well controlled in certain cases [72].

#### 4.2.1. Necrosis

Necrosis is an uncontrolled form of cell death induced by an external stimulus, such as inflammation or hypoxia [73]. This results in the expansion of organelles, plasma membrane fracture, and inflammatory responses induced by the leakage of intracellular contents [74]. Nevertheless, it involves different pro-inflammatory molecules, such as the dimeric nuclear factor-κB (NF-κB), a transcription factor [75]. Specifically, necrosis occurs in other forms [76,77]. Nevertheless, apoptosis can culminate in secondary necrosis in the absence of ATP [78].

#### 4.2.2. Apoptosis

Apoptosis is when cells cease to grow and multiply and enter a process mediated by cysteine proteases known as caspases [79] that ends in controlled death without spillage of its contents [73]. Generally, apoptosis can be initiated by intrinsic and extrinsic pathways [80], both described below. Specifically, it can be initiated through tumor necrosis factor (TNF) receptor type-1 [81] associated death domain (TRADD) [82], Fas-associated death domain (FADD) [83], and procaspase 8 [84,85].

##### Extrinsic Pathway

The extrinsic pathway of apoptosis is activated by the binding of TNF and the fibroblast-associated surface ligand (FasL) to the cell membrane’s death receptors [86], activating the death-induced signaling complex which, in turn, activates caspase-8 [87]. Specifically, Fas, a membrane-bound receptor that is part of the TNF superfamily, actuates the extrinsic apoptotic pathway through the crosslinking of FasL [88]. Moreover, procaspase-8 cleaves into caspase-8 and activates itself in an initiatory complex, thus inducing the extrinsic apoptotic pathway [84].

##### Intrinsic Pathway

The intrinsic pathway of apoptosis is controlled by the B-cell lymphoma-2 (Bcl-2) protein family [78]. This protein family divides into three subfamilies: anti-apoptotic, BH-3-only (proapoptotic), and pore-forming proteins (“executioners”; proapoptotic) [89]. Bax and Bak are proapoptotic proteins promoting the permeabilization of mitochondrial outer membrane [90]. Particularly, Bax/Bak insert themselves into the mitochondrial membrane, causing the subsequent release of cytochrome c into the cytosol, consequently combining with the oligomerization of the apoptotic protease activating factor-1 (Apaf-1) to create the apoptosome and, thus, activating caspase-9 [91,92].

## 5. Specific Candidate Peptides as Anticancer Therapeutics

Although cancer therapeutics have recently evolved, the evolution of cancer-site specific targeting peptides is still stagnant as the clinical field still awaits a molecule with the capacity of targeting a variety of cancers [93]. In this regard, many years have passed since the first anticancer peptide was found cytotoxic for various cell lines: magainin, from *Xenopus laevis* [94]. 

**Figure 1:**
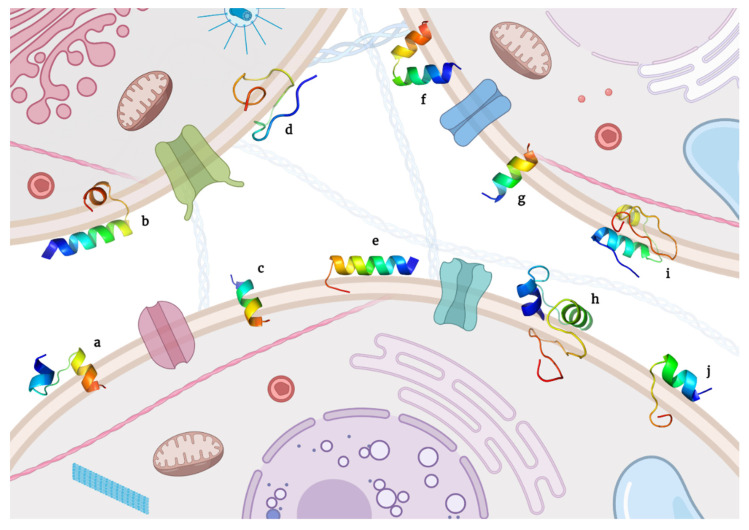
3D models of candidate peptides as anticancer therapeutics inducing membrane damage. (**a**) Buforin IIb, (**b**) ChMAP-28, (**c**) Decoralin, (**d**) Hepcidin isoforms TH1-5 and (**e**) TH2-3, (**f**) Magainin 2, (**g**) NaD1 defensin, (**h**) MP1, (**i**) Tachyplesin, and (**j**) Thionin.

**Figure 2:**
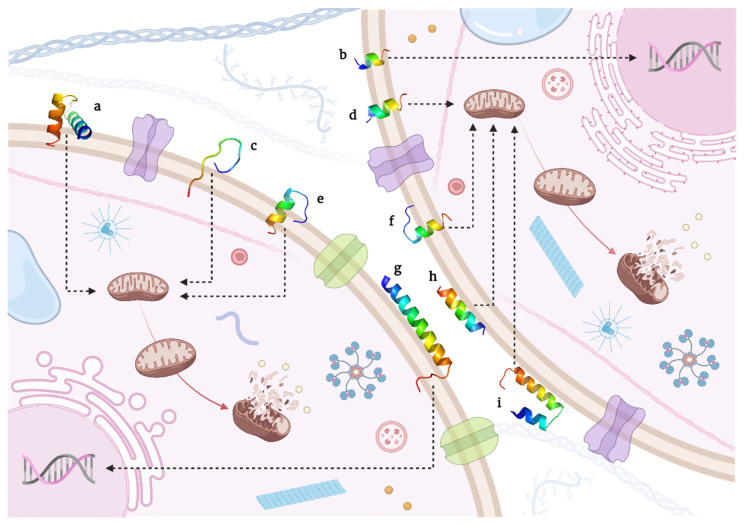
3D models of candidate peptides as anticancer therapeutics inducing apoptotic cell death. (**a**) Cecropin XJ, (**b**) *Cycas revoluta* peptide, (**c**) GG, (**d**) LF11, (**e**) Leucrocins KT2 and (**f**) RT2, (**g**) LL-37 native, (**h**) FK-16 fragment, and (**i**) Pardaxin.

This section discusses peptides having a known sequence and a specific recognized mechanism for inducing cancer cell death. Moreover, they are sorted in an arrangement of membrane damage and apoptotic cell death mechanisms. Correspondingly, their state-of-the-art, specific amino acid sequence (Table 1 and Table 2) and 3D models (Figure 1 and Figure 2) are presented. 

### 5.1. Peptides Performing Membrane-Damaging Cell Death

The effect on the cell membrane of peptides can be disruption, consequently resulting in cell lysis in a poorly controlled manner, resulting in the spilling of contents into the surrounding microenvironment (necrosis) [73]. Alternatively, their interaction with the membrane can form transient pores and then transport peptides inside cells, allowing them to interact with intracellular targets [95].

**Table 1:** Amino acid sequence of specific candidate peptides performing membrane damage.

Key	Peptide	Amino Acid Sequence	Reference
a	Buforin IIb	TRSSRAGLQFPVGRVHRLLRK	[96]
b	ChMAP-28	GRFKRFRKKLKRLWHKVGPFVGPILHY	[97]
c	Decoralin	SLLSLIRKLIT	[98]
d & e	Hepcidin isoforms TH1-5 and TH2-3	GIKCRFCCGCCTPGICGVCCRF & QSHLSLCRWCCNCCRSNKGC	[99,100]
f	Magainin 2	GIGKFLHSAKKFGKAFVGEIMNS	[101]
g	NaD1 defensin	ARECKTESNTFPGICITKPPCRKACISEKFTDGHCSKILRRCLCTKPC	[102]
h	MP1	ILGTILGLLKSL	[103]
i	Tachyplesin	KWCFRVCYRGICYRRCR	[104]
j	Thionin	KSCCRNTWARNCYNVCRLPGTISREICAKKCDCKIISGTTCPSDYPK	[105]

#### 5.1.1. Buforin IIb

Buforin IIb, a peptide derived from the histone 2A isolated from the Asiatic toad (*Bufo bufo garagrizans*), can translocate into the cytosol without membrane disruption then accumulate in the nuclei and, thereby, induce apoptosis [37]. Particularly, this peptide shifts itself through the plasmatic membrane through the formation of transient toroidal pores [106]. This peptide has induced cytotoxicity against breast, colon, and prostate cancer cell lines [50,107]. Nevertheless, the promising cytotoxicity of this peptide has been further improved with the production of a peptide with enhanced selectivity and with no toxicity for healthy cells, known as the BR2 peptide [108].

#### 5.1.2. ChMAP-28

ChMAP-28 is an antimicrobial peptide from the leukocytes of *Capra hircus* [109]. This peptide is selective to cancerous cells and is non-hemolytic, making it a promising peptide for oncological treatment [110]. Particularly, the ChMAP-28 peptide shows the capacity to provoke necrosis in cancer cells through plasmatic membrane disruption [7]. Several studies on its properties have discovered that this peptide resulted in cytotoxicity for A431 epidermoid carcinoma, HL-60 acute promyelocytic leukemia, and SKBR-3 human breast adenocarcinoma cells [97].

#### 5.1.3. Decoralin-NH2

Isolated from the venom of the *Oreumenes decoratus* wasp, decoralin-NH2 is another peptide that has demonstrated both antimicrobial and anticancer properties [111]. Specifically, decoralin-NH2 is capable of provoking necrosis through membrane micellization [112] in breast cancer (MCF-7) and sarcoma cells [7]. Reportedly, several analogs and modifications have been made to decoralin-NH2, which have shown similar anticancer activity but even less hemolysis [113,114].

#### 5.1.4. Hepcidin

Hepcidin is a peptide obtained from tilapia fish (*Oreochromis mossambicus*) [115]. Specifically, two of its isoforms, TH1-5 and TH2-3, have denoted cytotoxic effects [7]. TH1-5 changed the lipid membrane and induced necrosis in high concentrations and apoptosis in lower concentrations [116]. Moreover, TH1-5 and TH2-3 prevent cancer cell invasion through electrostatic interactions [7].

#### 5.1.5. Magainin 2

Magainin 2 is an amphiphilic α-helical membranolytic peptide obtained from the skin of the African frog (*Xenopus laevis*) [117]. This peptide also performs a synergistic antimicrobial activity [118]. The mechanism of action of magainin 2 is to rapidly induce ion channels causing leakage of ions such as Cl-, K+, and Na+ [119]. However, the cytotoxic effects of this peptide depend on the membrane potential [120]. Additionally, mitochondrial exposure to magainin 2 inhibits cellular respiration and leakage of glucose through peptide-induced channels [121].

#### 5.1.6. NaD1 Defensin

Defensins are a class of plant antimicrobial peptides resembling an anticancer mechanism that remains poorly understood [122,123,124,125,126]. NaD1 defensin has generated interest due to its already elucidated effect on monocytic lymphoma cells U937 [15]. Such a mechanism consists of cell lysis through direct binding to the phospholipid phosphatidylinositol 4,5-bisphosphate (PIP_2_) of plasmatic membranes [52,127].

#### 5.1.7. MP1 Peptide

The antimicrobial peptide MP1, isolated from a Brazilian wasp (*Polybia paulista*) [128,129], has selectively inhibited various tumor cell lines [130]. This peptide causes perturbation of the cell membrane in a two-sequence process: (1) union of the MP1 peptide to the plasmatic membrane, and (2) membrane disruption through bound peptides-induced leakage [131], making it a possible adjuvant for novel chemotherapeutic therapies [132]. Based on this, the positively charged nature of this peptide is likely a relevant factor for the first step, in which the peptide binds to the plasmatic membrane in a structured form, e.g., a helix [133,134]. Likewise, membrane permeabilization is part of the mechanism of cancer cell death mediated by this peptide [130,135,136].

#### 5.1.8. Tachyplesin

Tachyplesins are host defense peptides from horseshoe crabs (*Tachypleus tridentatus*) displaying antimicrobial and anticancer properties [137]. These peptides have indicated a high affinity for plasmatic membrane and selectivity against cancer cells, causing cell death by membrane disruption or apoptosis [138]. Additionally, there are reports of A549 adenocarcinoma human alveolar basal epithelial cells exhibiting resistance against this peptide, putting in doubt its true potential as an anticancer therapeutic [139]. Nonetheless, certain studies have indicated that this peptide has high selectivity for melanoma cells and high efficacy to internalize itself into cancer cells [138].

#### 5.1.9. Thionins

Interestingly, thionins were the first antimicrobial peptide obtained from plants [140,141]. Specifically, the thionin from mistletoe (*Pyrularia pubera*) reportedly has an anticancer effect, which has been attributed to a cellular response simultaneously triggering Ca^2+^ influx and depolarization of plasmatic membrane [142]. Notably, this peptide then activates endogenous phospholipase A_2_, subsequently triggering membrane disruption and, eventually, cell death [143].

### 5.2. Peptides Performing Apoptotic Cell Death

The term “apoptosis” refers to the termination of cells in a programmed manner involving a series of molecular events [144]. Recently, the termed proapoptotic peptides have risen as novel cancer therapeutics [145].

**Table 2:** Amino acid sequence of specific candidate peptides performing apoptotic cell death.

Key	Peptide	Amino Acid Sequence	Reference
a	Cecropin XJ	WKIFKKIEKMGRNIRDGIVKAGPAIEVLGSAKAIGK	[146]
b	*Cycas revoluta* peptide	AWKLFDDGV	[147]
c	GG	GPPPQGGRPQG	[148]
d	LF11	FQWQRNMRKVR	[149]
e & f	Leucrocins KT2 & RT2	NGVQPKYKWWKWWKKWW & NGVQPKYRWWRWWRRWW	[150]
g & h	LL-37 native and its FK-16 fragment	FRKSKEKIGKEFKRIVQRIKDFLRNLVPRTES & FKRIVQRIKDFLRNLV	[151,152]
i	Pardaxin	GFFALIPKIISSPLFKTLLSAVGSALSSSGGQE	[153]

#### 5.2.1. Cecropin XJ

Cecropin XJ, obtained from the larvae of silkworms (*Bombyx mori*), has reported activity against cancers, such as leukemia, gastric, esophageal, and hepatocellular (cell line Huh-7) cancers [7]. Specifically, cecropin XJ can cause apoptosis and inhibit cancer cell proliferation through the mitochondrial apoptosis pathways [154]. Moreover, cecropin XJ targets phosphatidylserine and phosphatidylethanolamine, both found in cancer cells’ outer membrane, and targets phosphatidylglycerol and cardiolipin, which explains its interactions with the mitochondria during apoptosis [155].

#### 5.2.2. *Cycas revoluta* Peptide

A small peptide with sequence AWKLFDDGV and a molecular mass of 1.050 KDa was obtained from palm fern seeds (*Cycas revoluta*) [147,156]. This peptide induced the inhibition of cancer cell proliferation by disrupting nucleosome structures, thus inducing apoptosis through DNA binding [15,157]. Through this mechanism, this peptide has exhibited clear adverse effects on colon carcinoma (HCT15) and human epidermoid cancer (Hep2) cells [158].

#### 5.2.3. GG Peptide

Human saliva has been one of the least studied sources of peptides. Nonetheless, research indicated that the coined GG peptide affects the capability of e-cadherin to stabilize adherent junctions, further causing apoptosis [159]. Moreover, the GG peptide induces the expression of the proapoptotic Bax protein, decreasing the Bcl-2/Bax ratio, thereby favoring apoptosis [160]. Thus, such results were considered promising for the potential use of saliva-derived peptides as therapeutic agents [161]. Nonetheless, further research must be conducted to better characterize salivary peptides as active against different cell lines and, thus, highlight the vast potential of these molecules [162].

#### 5.2.4. LF11 from Human Lactoferricin (hLFcin)

hLFcin comprises the 1–45 amino acid residues of the N-terminus of human lactoferrin (hLF) [163]. Particularly, LF11 is an 11-amino-acid fragment of hLFcin that has been optimized for its activity against cancer membranes [164]. To induce apoptosis, this peptide enters the cell through the PS, exposing sites on the cancer cell surface to then reach negatively charged targets on the surface of mitochondria, such as phosphatidylserine [165] and cardiolipin [166]. The relatively slow action, combined with the observation of membrane blebbing, is an indication of membrane-mediated apoptosis [167].

#### 5.2.5. Leucrocins

Isolated from crocodile leukocytes, leucrocin was engineered to improve its native features [168]. The most successful derivatives were KT2 and RT2 [150]. These peptides act as death ligands and upregulate death receptors such as TNF R1, Fas, and TRAIL R2 [7]. Because of the binding of these peptides, the FADD is activated, procaspase 8 is cleaved, the proapoptotic factor HTRA2 leaks from the mitochondria, and apoptosis ensues [169]. Furthermore, caspase-3, caspase-9, and Bax were significantly increased [170]. Likewise, studies have shown that KT2 and RT2 successfully inhibit colorectal, CaSki cervical, and HeLa cancer cells [171].

#### 5.2.6. LL-37 and Its FK-16 Fragment

The human antimicrobial protein of 18 kDa (hCAP18, mainly called LL-37) is the only cationic cathelicidin found in human secondary granules of neutrophils [172]. Specifically, it induces apoptosis through DNA fragmentation and mitochondrial depolarization, both independent of caspase activation [153], or through the intrinsic pathway [173]. Additionally, FK-16, a fragment of LL-37, induces apoptosis (caspase-independent) and autophagy through the common p53-Bcl-2/Bax pathway [174].

#### 5.2.7. Pardaxin

Pardaxin is a recognized peptide that was first isolated from the Red Sea Moses sole (*Pardachirus marmoratus*) [175]. The selective death induction of cancer cells has been attributed to its cationic net charge, which enables easy interaction with the anionic plasma membranes of cells [176]. The uptake of pardaxin into the cytosol allows it to bind to the mitochondria, which mostly contain phospholipids, phosphatidylethanolamine, phosphatidic acid, and cardiolipin [177]. This resulting cytochrome c leakage into the cytosol of HT-1080 cells after pardaxin treatment has been related to the pore-forming ability in mitochondrial membranes [178]. Likewise, the release of cytochrome c from the mitochondria into the cytosol results in the mitochondrion-mediated apoptotic pathway [179] and the activation of caspases 3/7 [180].

## 6. In Vivo Studies of Natural Specific Peptides

Until now, the majority of anticancer peptides has only been researched in vitro [181], thus one of the main limitations regarding the potential therapeutic applications of anticancer peptides is the scarcity of in vivo studies to support the results of in vitro experiments [182]. Precisely, this is the reason why this review addressed specific candidate peptides at such a research phase. The literature reports on hydrolysates from different sources containing peptides with known sequence [183].

A leading representative of natural peptides with recognized anticancer activity is lunasin [184]. This is a peptide composed of 43 amino acid residues and isolated from soybeans [185] and is recognized as containing the arginine-glycine-aspartic acid (RGD) cell adhesion motif located at its carboxyl end [186]. Interestingly, this motif preferentially binds to deacetylated histone H4 in vitro while, in vivo, inhibits histone H3 and H4 acetylation [187]. Moreover, lunasin increases apoptosis and inhibits caspase-3 both in vitro and in vivo [188].

There are additional peptides with anticancer effects tested in vivo [189,190]. However, their structure is more complex than the candidates addressed in this review. In this regard, a remarkable polypeptide is Vglycin, a 37-residue purified from pea seeds [191]. Its capability to inhibit colon cancer growth in vivo was an important finding, as well as its mitochondrial swelling and nuclear chromatin condensation, thereby denoting it as a cancer therapeutic agent [192].

## 7. Disadvantages of Peptides Targeting Cancer Cells

Even though numerous anticancer peptides have been thoroughly described and their activity has been demonstrated, their use may be limited by their rapid kidney and liver clearance [193], protease degradation by hydrolysis [194], or instability in gastrointestinal tract [195] or body fluids, such as blood [196].

Due to these intrinsic limitations, some bioactive peptides usually undergo modifications, such as the use of D-aminoacid or unnatural aminoacids [50]. Further proposals for improvements of peptides in the body’s environment include C- and N-terminal modifications, pegylation, post-translational modifications, such as glycosylation, and creating cyclized or stapled peptide structures to enhance biostability and blood circulation time [93,197].

## 8. Discussion

As the cancer epidemic is still on the rise [198], a strategy to expand the options for cancer treatment is therapeutic peptides. While its definition remains in constant debate [17], the role of this research in this field is to focus on peptides already considered as drug candidates due to their remarkable results in nonclinical tests.

Herein, the level of knowledge of the specific pathways or mechanisms of action of such peptides was analyzed, given that both results are essential to move toward clinical trials. Moreover, the focus was specifically on peptides with a known amino acid sequence since there are many extracts and protein fractions with reported anticancer effects but without a clear molecular protagonist.

Particularly, this is due to the anticancer mechanism peptides, which vary from current traditional anticancer drugs [26]. In this regard, and very interestingly, among the peptides with damaging effects on the cell membrane, many of them denoted antimicrobial and anticancer effects which, accordingly, agreed with the non-specificity of the charge interaction mechanism. Specifically, peptides such as ChMAP-28 or decoralin-NH_2_ exhibited a broad spectrum of cancerous cell lines among their targets [7,97].

In contrast, studies involving different cell lines have exhibited a certain resistance to the effects of tachyplesins [139]. This drawback for clinical development is consonant with multiple in vitro and in vivo studies with promising results [199]; a disadvantage regarding those peptides is the induction of more targeted cell death. For instance, among the peptides with recognized mechanisms of apoptosis induction presented here, most of them have a broad capability of causing cell death by targeting mitochondria. This approach implies intervention in the behavior of cancerous cell mitochondria, thereby avoiding the spread of potentially mutated mitochondrial DNA and metastasis [200].

Moreover, certain candidates, such as cecropins or leucrocins, have been engineered to enhance their notable activities against cancer cells [150,201]. This is a current trend in which bioinformatic algorithms combine with machine learning, which is currently considered the future for the rational design of peptides [202]. In response, the synthesis of specific peptides based on previous in silico prediction and design would effectively reduce the time for the obtention of more reproducible and function-specific peptides.

Previously, specialists have already been encouraged to use this approach as part of their routine activities for peptide design [203]. So far, the strategy has resulted in remarkable data on its performance and applicability. For instance, the buforin IIb peptide, which, to our knowledge, is in its enhanced version of BR2 peptide, deserves to be considered for more advanced nonclinical testing based on its confirmed selectivity and null toxicity for healthy cells [108]. Additionally, the reports of certain peptides interacting with overexpressed plasmatic proteins, such as ATP-binding cassette subfamily B member 1 (ABCB1) [204], or displaying a more selective effect against specific cancer types, mainly attributed to the differences in membrane composition and electrochemical properties [46], point to a path that has not been fully considered for the improvement of new candidate peptides.

Generally, the intrinsic weaknesses of these peptides should be overcome with the aid of protein engineering, attempting to avoid poor chemical and physical stability, short circulating plasma half-life, and enzymatic degradation [19,21].

Furthermore, crescent technologies such as D-enantiomeric or peptide stapling can improve the similar-to-drugs features [205]. Likewise, efforts should be made to improve the capacity of many of these peptides to be innocuous to healthy cells and immune to cell resistance mechanisms, as it would represent notable progress for cancer treatment [23]. For instance, a targeted activation when approaching the vicinity of cancer cells, based either on relatively acidic pH or on cleavage by metalloproteinases [206]. Finally, studies should be extensive to consider novel peptides’ roles as companions to recognized anticancer drugs, e.g., chemosensitizers [207].

Thus, the coming years will provide answers to whether this strategy results in large-scale use of these candidate peptides.

## 9. Conclusions

Although peptide relevance as cancer therapeutics has increased over the years, the number of peptides with recognized sequence and specific mechanisms of action against cancer cells is still limited. Moreover, an essential part of these peptides relies on the non-specific mechanism of damaging the cell membrane. Thus, for now, appropriate recommendations should be to overcome the intrinsic weaknesses of instability in body fluids, kidney clearance, and protease degradation of candidate peptides, improve their sequences with unnatural amino acids or post-translational modifications, modify their chain terminal ends, create stapled structures to enhance their biostability and blood circulation time, as well as to use recognized mechanisms of apoptosis induction and enhance their capacities of non-toxicity to healthy cells and further avoid cell resistance.

## Data Availability

Not applicable.
